# Social motor synchrony in autism spectrum conditions: A systematic review

**DOI:** 10.1177/13623613231213295

**Published:** 2023-11-28

**Authors:** Devyn Glass, Nicola Yuill

**Affiliations:** University of Sussex, UK

**Keywords:** autism spectrum conditions, interpersonal synchrony, social motor synchrony

## Abstract

Some researchers suggest difficulties synchronising with a partner could underpin the social differences associated with Autism Spectrum Condition, potentially acting as a marker for autism. Social Motor Synchrony (SMS) is one aspect of synchrony that could augment observational diagnostic procedures. However, the full breadth of literature examining SMS in autism has not been systematically reviewed. A systematic review following the Preferred Reporting Items for Systematic Review and Meta-Analysis guidelines yielded 29 papers meeting inclusion criteria. Of 21 studies including a comparison group, all indicated weaker SMS between two autistic partners and in mixed-neurotype partnerships compared with two non-autistic partners. Papers involving mixed-neurotype pairs without a comparison group showed higher than chance SMS and demonstrated an increase in SMS over time following rhythm-based interventions. Although research so far demonstrates weaker SMS in pairs involving an autistic partner compared with non-autistic pairs, we identified several limitations which may have influenced SMS in autistic people and their partners. Further work is needed with autistic partnerships, more natural and preferred tasks within comfortable settings and partnerships, and more work to identify the basis of synchrony differences. We set out implications for design of further research.

**Lay abstract:**

When two people interact, they often fall into sync with one another by moving their bodies at the same time. Some say autistic people are not as good as non-autistic people at moving at the same time as a partner. This has led some researchers to ask whether measuring synchrony might help diagnose autism. We reviewed the research so far to look at differences in Social Motor Synchrony (SMS) (the way we move together) between autistic people and people they interact with. The research suggests that interactions involving an autistic partner (either two autistic partners, or an autistic and non-autistic partner) show lower synchrony than a non-autistic pair. However, we recognised elements in the research so far that may have affected SMS in interactions involving an autistic person. One way SMS may have been affected in research so far might be the way interactions have been set up in the research studies. Few papers studied interactions between two autistic people or looked at synchrony in comfortable environments with autistic-preferred tasks. The studies also do not explain why synchrony might be different, or weaker, in pairs involving autistic partners. We use these limitations to suggest improvements for future research.

Interpersonal synchrony is a quality of a relationship with a key role in social interaction; facilitating the smooth flow of social exchanges and helping to forge social bonds (Bernieri & Rosenthal, 1991; Tuncgenc and Cohen, 2016). It is involved in a range of socially beneficial behaviour, notably in relationship development. It predicts feelings of affiliation and rapport, which are important for the development of social relationships and feelings of connectedness (Hove & Risen, 2009; Miles et al., 2009; Rabinowitch & Knafo-Noam, 2015). Interpersonal synchrony in Autism Spectrum Conditions (ASC) is a research area with growing interest, owing to differences from non-autistic people in social communication and interaction, which some argue may be related to differences in interpersonal synchrony (e.g., Fitzpatrick et al., 2017a).

Bernieri and Rosenthal (1991) refer to interpersonal synchrony as the smooth meshing of simultaneous and rhythmic activity of two or more individuals, which occur at the same time or at the same rate. This includes the coordination or matching in time of a range of human social behaviour, such as conversational alignment, imitation, shared effect, and several non-verbal behaviours including physiological synchrony or the synchronisation of motor movements (Bernieri & Rosenthal, 1991; Charman, 2011). Socially synchronous behaviours need not be identical or simultaneous but are rhythmically matched or related in time (Valdesolo et al., 2010). Interpersonal synchrony therefore includes, for example, simultaneous behaviours, such as rhythmically matched body movements or expressions of affect (Chartrand & Lakin, 2012), and behaviours with an extended temporal sequence, as seen in reciprocal or contingent behaviours, such as raising one’s hand to meet a partner’s handshake (Feldman, 2007). Social interaction is a dynamic and time-unfolding process, and various interrelated behaviours contribute to ease and efficiency in social interaction.

Interpersonal synchrony is seen in human behaviour from infancy and is thought to be related to the development of language and social communication (Condon & Sander, 1974). In addition, synchronised movement is an important contributor to successful collaboration, itself a vehicle for developing social and cognitive abilities (Moll & Tomasello, 2007; Vygotsky, 1978). Interpersonal synchrony is an embodied way to achieve engagement and intersubjectivity, and thus collaboration (De Jaegher et al., 2017; Prepin & Pelachaud, 2013). The way we move together therefore stimulates social connection and engagement in the types of interaction that build stronger social relationships and gives rise to advanced socio-cognitive skills (Miles et al., 2009). Since social interactions with others are embodied, and interpersonal synchrony is implicated in a range of socially beneficial behaviours, then lower interpersonal synchrony may be associated with differences or disruptions to social interaction (Fitzpatrick et al., 2013). Some research indicates that interpersonal synchrony is less frequent in interactions where one or more partners has a condition that typically affects social interaction, such as autism or schizophrenia (e.g., Fitzpatrick et al., 2017b; Ramseyer & Tschacher, 2011).

Autism is a diverse neurodevelopmental condition characterised by differences from non-autistic people in social communication and interaction, as well as in cognitive flexibility, and sensory needs (American Psychiatric Association, 2013). These differences manifest in markedly different ways between individuals, but differences in social interaction compared with non-autistic people can sometimes make everyday interaction challenging (Crompton, Ropar, et al., 2020; Pennington et al., 2014). Fitzpatrick et al. (2017b) found that interactions involving an autistic partner and a non-autistic partner display less Social Motor Synchrony (SMS) than interactions between two non-autistic people. SMS is a distinct element of interpersonal synchrony, referring to synchronous motor movements within social interaction. SMS is concerned solely with body movements, rather than other aspects of interpersonal synchrony, such as physiological synchrony or behaviours that indicate mutual engagement or shared affect, such as facial expressions or joint attention (Fitzpatrick et al., 2016). It therefore comprises non-verbal and non-facial motor synchrony. Some studies demonstrate lower SMS when one partner is autistic than between two non-autistic partners (e.g., Fitzpatrick et al., 2017b; Georgescu et al., 2020). In addition, performance on tasks tapping SMS correlates with various measures of social competence and clinical measures of autism (Fitzpatrick et al., 2017a) and increases in dyadic attunement and synchrony can lead to greater attentiveness and social initiation in autistic children (Green et al., 2017). Consequently, some researchers subscribe to an SMS model of autism whereby differences in the tendency to synchronise motor movements may underlie some of the social differences associated with autism (Fitzpatrick et al., 2016).

Research has begun to explore the potential for using SMS in the autism diagnostic process. Koehler et al. (2022) demonstrated weaker SMS in interactions involving individuals who go on to receive an autism diagnosis compared with those who did not receive one. However, the process of engaging in synchrony with a partner is complex, and Loth et al. (2021) emphasised that for biomarkers to have diagnostic utility, prominent and consistent group differences are needed. This presents a potential issue for the use of SMS as a diagnostic marker as the heterogeneity of experiences and challenges in autism are widely recognised. Autistic people are often described as having ‘spikey profiles’, with strengths in some areas and difficulties in others (Frith, 1996). SMS also involves several additional cognitive processes. For instance, partners are required to integrate social information from several modalities (Bowsher-Murray et al., 2022). Multisensory integration itself can be a challenge for some autistic people, which could act as a contributing factor to difficulties with interpersonal synchrony (Collignon et al., 2013). A systematic review of the SMS literature would therefore enable an overview of current SMS literature. A review will also allow identification of elements of synchrony tasks that may specifically influence SMS in autistic people and their partners, which will support careful integration of SMS into diagnostic assessments.

There are several aspects of interpersonal synchrony, and some existing reviews have considered SMS together with other interpersonal synchrony constructs, such as conversational, neural, or audio-visual synchrony (Baldwin et al., 2022; Mcnaughton & Redcay, 2020). McNaughton and Redcay (2020) demonstrated that findings across synchrony domains do not necessarily align. For instance, they found several papers demonstrating similar conversational alignment in autistic and non-autistic participants, but they found weaker synchrony in mixed-neurotype pairs compared with non-autistic pairs in motor, physiological and neural synchrony. SMS is a specific, well-defined aspect of interpersonal synchrony with an increasing amount of research. However, existing reviews include limited papers relating to SMS, and McNaughton and Redcay (2020) do not describe a systematic approach, meaning the full breadth of SMS research has so far not been synthesised. Parsing the different components of interpersonal synchrony into discrete reviews will enable a more thorough investigation into potential biomarkers for autism.

This article is the first to systematically review the literature on SMS in autism. Our primary aim is to determine whether SMS differences are apparent between autistic, non-autistic and mixed-neurotype partnerships. We also aimed to identify methodological limitations and gaps in the current literature, to further our theoretical understanding of SMS in autism and to support potential practical applications of SMS research in diagnosis and intervention.

## Method

We followed the Preferred Reporting Items for Systematic Review and Meta-Analysis (PRISMA) checklist (Page et al., 2021) and pre-registered a protocol following the PRISMA-P guidelines (Shamseer et al., 2015). See PROSPERO international prospective register of systematic reviews (registration number: CRD42019119480).

### Selection criteria

Studies were eligible for the review if they: (a) empirically assessed temporally matched, non-verbal and non-facial body movements during social interaction with another person or agent, (b) involved participants with an Autism or Asperger’s Syndrome diagnosis, and (c) were published in English in peer-reviewed academic journals. We excluded non-English papers, as expert translation was unavailable to us. We also excluded studies if they: (a) only investigated intentional imitation or mirroring behaviours without a measure of temporal coordination, (b) did not comprise a full-length original report, such as reviews or commentaries, (c) measured or analysed according to autistic traits, not by diagnosis, (d) if they did not specify the dyad for whom they report a synchrony score (e.g., for interactions involving triads or groups), and (e) only assessed physiological synchrony, such as electrodermal activity or heart rate. While social synchrony correlates positively with physiological synchrony, such synchrony can occur in the absence of social interaction (Feldman et al., 2011; Konvalinka et al., 2011). There were no restrictions according to study design or setting. Randomised Control Trials (RCTs) were eligible, providing they empirically measured SMS; RCTs that contained elements of synchrony within an intervention, but which were not empirically measured, were not eligible.

### Search strategy

We performed an electronic search up until April 2022 across five databases: SCOPUS, Web of Science, PsycArticles, PsycINFO, and PubMed. Search terms relating to ASC were paired with search terms for SMS using Boolean operators. Where possible, we imposed limits on the search to narrow the subject area and to exclude common, irrelevant key words. The full search string for each database can be found in the Supplementary Material (Appendix 1). We manually examined reference lists from identified papers and from topic-relevant papers to find papers not identified through database searching. We sought unpublished works that met the inclusion criteria by searching Open Access Dissertations and Theses (OATD) and EThOS, from the British Library.

### Data screening

**Figure 1.**
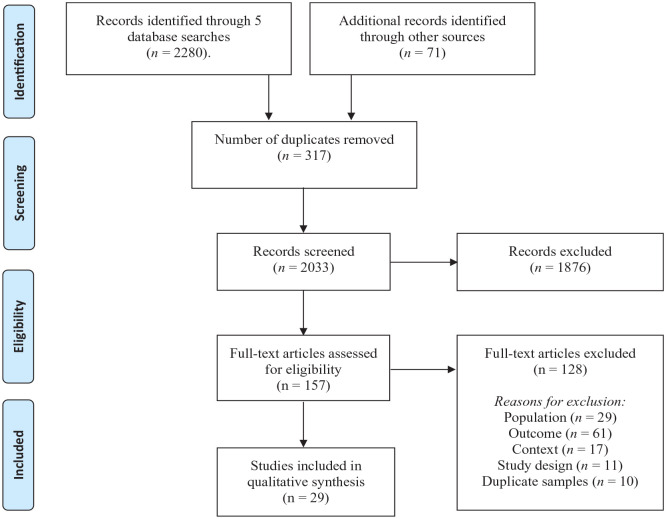
PRISMA flow diagram.

We conducted the review in two stages. At each stage, both authors independently reviewed the articles; we reviewed any conflicts and resolved them through discussion until we reached 100% agreement. First, following the removal of any duplicate papers (*n* = 317), all references yielded from the initial database searches (*n* *=* 2280) and screening of the additional resources and grey literature (*n* = 71) were collected in the review software Rayyan, a web application designed for title and abstract screening (Ouzzani et al., 2016). Both reviewers independently screened all titles and abstracts to identify relevant papers (*n* *=* 157). Second, each reviewer independently conducted a full-text review, which resulted in a final set of 29 articles (see Figure 1). We excluded papers if the same data had been reported elsewhere.

### Data extraction and analysis

The first author manually extracted the core information from the final set of articles, including the aims, participant characteristics and diagnoses, dyadic relationship, study design, details of tasks and measurements, conceptualisation and measurement of synchrony, and main results. To summarise the findings, we divided the articles into three groups. The first group involved studies with adult participants (*n* = 5). The second group involved child participants (*n* = 24), one of which included two studies with distinct samples, so were included as two separate studies (Yoo & Kim, 2018). Group 3 included one study that involved infants younger than 18 months (Yirmiya et al., 2006). They would not have undergone diagnostic assessments for autism, so were a separate category.

### Risk of bias

As recommended in the PRISMA guidelines, we assessed the risk of bias for each study (Page et al., 2021). The first author assessed study quality and the second author independently rated 25% of the papers. We used the ‘Quantitative Descriptive Studies’ scale from the Mixed Methods Appraisal Tool (MMAT) to assess study quality and risk of bias, which describes the existing distribution of variables (Grimes & Schulz, 2002; Hong et al., 2018). The MMAT discourages an overall quality score, favouring an in-depth presentation of judgements about sampling strategy, representativeness, measurements and analyses and non-response bias. The initial percentage of agreement to the MMAT was 93%. Papers were discussed until consensus was reached. Both reviewers’ elaborations determined the limitations of the studies included in the review, which we used to generate a critical summary of the findings.

## Results

### Study characteristics

#### Participants

Table 1 provides a summary of participants’ demographics, combined according to participant group. Not all studies with autistic participants included a comparison group. Those that did (*n* = 22) mostly included pairs of non-autistic partners as a comparison group; however, one study compared autistic children and their therapy dog to children with Down’s syndrome and their therapy dog (Griffioen et al., 2020), and two studies compared across three groups. First, Xavier et al. (2018) compared autistic children, non-autistic children, and children with Developmental Coordination Disorder (DCD) interacting with a virtual partner. Second, Georgescu et al. (2020) compared all three dyad types: autistic, non-autistic, and mixed-neurotype pairs. The remaining seven papers lacked a comparator, and examined SMS during a standalone drama performance (Ward et al., 2018), assessed changes in SMS following an intervention (Dvir et al., 2020; Koehne et al., 2016; Manders et al., 2021; Venuti et al., 2017; Yoo & Kim, 2018), or examined SMS compared with chance (Romero et al., 2018).

**Table 1.** Demographics of included studies.

	Autistic adults		Autistic children/adolescents			Infants	
	ASC participants	NT participants	ASC participants	NT participants	Other participants	who have autistic sibs	who have NT Sibs
Number of Studies	6	5	24	17	2 (1 DCD; 1 DS)	1	1
Number of Participants							
Mean	22	28	18	23	11	51	52
Minimum	16	23	1	7	5	51	52
Maximum	34	35	45	53	17	51	52
Total	134	111	423	384	22	51	52
Age (years)							
Mean	22	33	22	7	13	0.82	0.82
Missing Data	–	–	1	–	–	–	–
Gender (%)							
Male	69%	58%	74%	71%	59%	63%	60%
Female	31%	42%	26%	29%	41%	37%	40%
Missing Data	–	–	1	–	–	–	–

ASC: Autism Spectrum Conditions; DCD: Developmental Coordination Disorder; DS: Down’s Syndrome; NT: Neurotypical; Sibs: Siblings.

There were 15 variations of relationship types in the included papers (e.g. child-clinician, or adult-expert improviser), summarised in Table 2. Most studies compared mixed dyads (an autistic and non-autistic partner) and non-autistic dyads (*n* = 16). Two of the four adult studies used this design and the children’s studies mostly did so (*n* = 14). Only three studies included autistic dyads; Georgescu et al.’s (2020) study, a study comparing pairs of autistic children to pairs of non-autistic children (Stoit et al., 2011), and a group interaction study, which involved autistic peer interactions, as well as interactions between autistic children and non-autistic facilitators (Ward et al., 2018). Three studies compared autistic and non-autistic children or adults and virtual partners (Kawasaki et al., 2017; Kostrubiec et al., 2018; Xavier et al., 2018). One study compared autistic children and their therapy dog with children with Down’s syndrome and their therapy dog (Griffioen et al., 2020). The final study with a comparison group included a parent and their infant who had an older autistic sibling (SIBS-ASC) compared to a parent and their infant who had a non-autistic older sibling (SIBS-NT; Yirmiya et al., 2006). The remaining seven studies considered mixed pairs without a comparison group (Dvir et al., 2020; Griffioen et al., 2020; Koehne et al., 2016; Manders et al., 2021; Romero et al., 2018; Venuti et al., 2017; Yoo & Kim, 2018).

**Table 2.** Procedures used to investigate synchrony in ASC and by autistic traits.

Study	Type of SMS	Task	Measurement of SMS	Diagnosis confirmed	Other measures	Type of dyad	Relationship of dyad	Results
*Adults*								
Brezis et al. (2017)	Spontaneous	The Mirror Game: Joint Improvisation	Percentage of co-confident (CC) motion segments	Previous diagnosis ADOS-2	SRS-2, WASI, Imitation battery, RBS-R; RME; TEQ, TAS-20; PANESS test, Florida Apraxia Battery and motor coordination	ASC-NT NT-NT	Adult participants & ‘expert improviser’	NT-NT > ASC-NT
Georgescu et al. (2020)	Spontaneous	Conversation	Windowed cross-lagged correlations using time series data from Motion Energy Analysis	Previous diagnosis Clinical interviews using ICD-10 criteria	AQ, EQ, SQ, TAS20, BDI, WST (German Verbal IQ)	ASC-NT NT-NT ASC-ASC	Two unfamiliar adult participants	NT-NT > ASC-ASC > ASC-NT
Kawasaki et al. (2017)	Intentional	Tapping task with human partner or virtual agent	Reaction time coherence	Previous diagnosis ADOS MSPA	AQ, IQ, VIQ, PIQ, WISC-3/WAIS-3/WAIS-R	ASC-NT NT-NT ASC-virtual NT-virtual	Two unfamiliar adult participants	Human: NT > ASC Virtual: NT > ASC
Koehler et al. (2022)	Spontaneous	Diagnostic assessments	Cross correlations computed from motion energy data	Diagnostic interviews AQ	EQ, ADC, BDI, SQ, TAS-20, WST	ASC-NT NT-NT	Adult participants and diagnostician	NT-NT > ASC-NT
Koehne et al. (2016)	Intentional	Dance and Movement Therapy	Assessment of Spontaneous Interaction in Movement video coding; orientation of gaze and body towards confederate, relation in spatial movement, imitation/synchronisation, and reciprocity/dialogue in movement	Previous diagnosis ADOS-2 ADI-R	IRI, MET, Automatic Imitation paradigm, asynchrony of finger tapping to a virtual partner	ASC-NT	Adult participants and an experimental confederate	Post Intervention > Pre-Intervention
*Children*								
Delaherche et al. (2013)	Spontaneous	3D Puzzle: Joint Action (JA) Task	Cross correlations computed from motion energy images	Previous diagnosis	VABS-2 or PEP-3, pragmatic conversation skills	ASC-NT NT-NT	Child participants and unfamiliar therapist	NT-NT > ASC-NT
Dvir et al. (2020)	Spontaneous	Music Therapy	Tension Flow Rhythm category from KMP; shared segments identified	Previous diagnosis ADOS-2 ADI-R	K-ABC, Cognitive Functioning estimated	ASC-NT	Child participants and music therapist	Post Intervention > Pre-Intervention
Fitzpatrick et al. (2013)	Intentional	SMS Task Battery	Relative phasing between two time series captured via motion energy analysis	Previous diagnosis	Developmental Profile 3, ToM task, JA tasks, intentionality tasks, cooperation tasks, imitation, manual motor dexterity	ASC-NT NT-NT	Child participants and experimenter	NT-NT = ASC-NT (all conditions aside from ‘object-object’)
Fitzpatrick et al. (2016)	Intentional & Spontaneous	Pendulum Paradigm	Relative phasing between two time series captured via motion energy analysis	Previous diagnosis ADOS-2	WASI vocabulary matrix and IQ matrix	ASC-NT NT-NT	Child participants and their parents	NT-NT > ASC-NT (Spontaneous looking & Intentional) NT-NT = ASC-NT (Spontaneous looking away)
Fitzpatrick et al. (2017a)	Intentional & Spontaneous	SMS Task Battery and Interpersonal Handclapping	Relative phasing between two time series captured via motion energy analysis	Previous diagnosis ADOS-2	CELF-4, DAS-2, Motor control tasks	ASC-NT NT-NT	Child participants and experimenter	NT-NT > ASC-NT
Fulceri et al. (2018)	Spontaneous	Joint Action (JA) Tasks (reaching and grasping)	Weighted coherence of the time series gathered using accelerometers	Previous diagnosis ADOS-2	SCQ (to screen NT participants), WPPSI, WISC-4	ASC-NT NT-NT	Child participants and experimenter	NT-NT = ASC-NT (clear end-point) NT-NT > ASC-NT (unclear end-point)
Griffioen et al.(2020)	Spontaneous	Dog-Assissted Therapy (DAT)	Manual coding of movement transformed into time series, which were then subject to Cross Recurrence Quantification Analysis to capture behavioural matches	Previous diagnosis	CBCL	ASC-animal DS-animal	Child participants and therapy dog	ASC-animal > chance Post Intervention > Pre-Intervention
Kaur et al. (2018)	Intentional	Motor Coordination Experimental Paradigm	Manual coding of movements for child and adult. Synchrony determined by coding start and end of synchrony periods	Previous diagnosis ADOS-2	SBIQ, SIPT-BMC, BOT-2,	ASC-NT NT-NT	Child participants and experimenter	NT-NT > ASC-NT
Kostrubiec et al. (2018)	Intentional & Spontaneous	Computerised Coordination Task–Oscillating Dot Paradigm	Cross correlations of continuous relative phase patterns captured using potentiometers	Previous diagnosis ADI-R	VABS-2, SCQ, WISC-4, DCDQ	ASC-Virtual NT-Virtual	Child participants and virtual partner	NT-NT = ASC-NT (Spontaneous) NT-NT > ASC-NT (Intentional)
Liu et al. (2022)	Spontaneous	Book sharing activity	Cross correlations from timeseries analyses, extracted from video data using a frame differencing method	ADOS-2	MSEL, VR, RL/EL	ASC-NT NT-NT	Child participants and their (presumed NT) caregiver	NT-NT > ASC-NT
Manders et al. (2021)	Spontaneous & Intentional	Dance and Movement Therapy	Judgements by naïve coders on a 4-point Likert scale, and qualitative descriptions	Previous diagnosis	Distraction and sensory seeking behaviours rated on 3-point Likert scales; interaction quality rated on a 4-point Likert scale	ASC-NT	Adolescent/adult participants and DMT therapists	Open segments > leading/following segments; differences with different partners
Marsh et al. (2013)	Spontaneous	Rocking Chair Paradigm	Cross correlations of continuous relative phase patterns captured using motion trackers	Previous diagnosis ADOS-2	MSEL	ASC-NT NT-NT	Child participants and their parents	NT-NT = ASC-NT (anti-phase synchrony) NT-NT > ASC-NT (in-phase synchrony)
Noel et al. (2017)	Spontaneous	Conversation	Cross correlations computed from motion energy data	Previous diagnosis ADOS-2 ADI-R	CELF; TONI-4–word judgement, Woodcock Johnson 3–cognitive ability, Motor Behaviour	ASC-NT NT-NT	Child participants and experimenter	NT-NT > ASC-NT
Romero et al. (2016)	Intentional & Spontaneous	Object Tapping (SMS Battery) and Interpersonal Handclapping	Relative phasing between two time series captured via wrist motion sensors	Previous diagnosis ADOS-2	N/A	ASC-NT NT-NT	Child participants and experimenter	NT-NT > ASC-NT
Romero et al. (2018)	Spontaneous	Conversation	Cross wavelet analyses from two time series captured via motion energy analysis	Previous diagnosis ADOS-2	JA, ToM, CELF, DAS-2	ASC-NT	Child participants and unfamiliar clinician	ASC-NT > chance
Stoit et al. (2011)	Intentional	Joint Digital Balancing Tasks	Reaction time coherence	Previous diagnosis ADOS-2 ADI-R	WISC-3, CBCL, VISK	ASC-ASC NT-NT	Two unfamiliar child participants	NT-NT > ASC-ASC
Su et al. (2021)	Intentional	Whole body-sway synchrony task	Cross-spectral analysis from Motion tracking data	Previous diagnosis	SCQ, VABS-2, SRS-2, ICS, BOT-2	ASC-NT NT-NT	Adolescent participants and adult tester	NT-NT > ASC-NT
Venuti et al. (2017)	Spontaneous	Improvisational Music Therapy (IMT)	Observational coding of child/adult behaviours, combined to give a synchrony score	Previous diagnosis ADOS-2 *DSM*-IV-TR assessment	Griffiths Mental Development Scales	ASC-NT	Child participants and music therapist	Post Intervention > Pre-Intervention
Ward et al. (2018)	Spontaneous	Theatre Performance–multi-person interaction	Cross wavelet analyses of participants wrist movements captured via wrist worn accelerometers	Previous diagnosis	N/A	ASC-NT ASC-ASC	Multi-person interaction including autistic peers and NT drama facilitators	Qualitative description of SMS between children, actors & music
Yoo & Kim (2018) *Study 1.*	Intentional	Dyadic Drumming	Asynchrony between the onset timing of tapping and the onset of the cueing	Previous diagnosis	K-WISC-4, K-CARS, K-SSRS, Imitation tasks, facial expressions, KDEF	ASC-NT NT-NT	Child participants and experimenter	ASC-NT w/ metronome > ASC-NT w/ interpersonal drumming
Yoo & Kim (2018) *Study 2.*	Intentional	Dyadic Drumming (following intervention)	Asynchrony between the onset timing of tapping and the onset of the cueing	Previous diagnosis	K-CARS, K-SSRS, Imitation tasks, Eye Gaze	ASC-NT	Child participants and music therapist	Post Intervention > Pre-Intervention
Xavier et al. (2018)	Intentional	Tightrope Walker Paradigm	Correlation between the participants’ bar angles and tightrope walkers’ bar angles	Previous diagnosis ADOS-2 ADI-R	WISC-4, M-ABC	ASC-Virtual NT-Virtual	Child participants and virtual partners	NT & DCD > ASC
Zadok et al. (2022)	Spontaneous	Conversation/ Confederate distress	Global judgements by naïve coders, rated in a 5-point Likert scale	Previous diagnosis ADOS-2	SRS-2, WISC-4, WAIS-3 (Hebrew versions, vocabulary subsets), SSIS-SS,EQ	ASC-NT NT-NT	Adolescent participants and confederates	NT-NT > ASC-NT
Zampella et al. (2020)	Spontaneous	Conversation	Behavioural rating of movement, focusing on form and timing	Previous diagnosis ADOS-2	SCQ, WASI, WISC-4, SRS-2 (to rule out ASC symptoms in Mothers), SSIS-RS, CCC-2	ASC-NT NT-NT	Child participants and research assistant or their mothers	NT-NT > ASC-NT
*Infants* *<* *3 years*								
Yirmiya et al. (2006)	Spontaneous	Free-Play Interactions	Cross correlations of time series: measurement of the time series data unclear	Sibling with autism diagnosis	ESCS, CHAT, Still Face Paradigm, Responsiveness to name calling, Gaze and Affect, BSID-2, ICQ.	SIBS(A)-NT SIBS(NT)-NT	Child participants and their mothers	SIBS-NT > SIBS-ASC

ADC: Adult Developmental Coordination Disorders/Dyspraxia Checklist; ADOS: Autism Diagnostic Observation Scale; AQ: Autism Spectrum Quotient; ASIM: Assessment of Spontaneous Interaction in Movement; BDI: Beck Depression Inventory; BOT-2: Bruininks-Oseretsky Test of Motor Proficiency–2nd Edition; CBCL: Child Behaviour Checklist; CCC:2 Children’s Communication Checklist, Second Edition; CELF-4: The Clinical Evaluation of Language Fundamentals–4; CHAT: Checklist for Autism in Toddlers; DAS-2: Differential Abilities Scales 2nd Edition; DCDQ: Developmental Coordination Disorder Questionnaire; DMT: Dance and Movement Therapy; EQ: Empathy Questionnaire; ESCS: Early Social Communication Scales; ICS: interpersonal communication scale; IJS: Interpersonal Judgement Scale; IRI: Interpersonal Reactivity Index; K-ABC: Kaufman Assessment Battery for Children; K-CARS: Korean Childhood Autism Rating Scale; K-SSRS: Korean Social Skills Rating System; K-WISC-4: Korean Wechsler Intelligence Scale for Children-4; KDEF: Karolinska Directed Emotional Faces; KMP: Kestenberg Movement Profile; M-ABC: Movement Assessment Battery for Children; MASC: Movie for the Assessment of Social Cognition; MET: Multifaceted Empathy Test; MSEL: Mullen Scales of Early Learning; MSPA: Multi-dimensional Scale for Pervasive developmental disorder and Attention deficit/hyperactivity disorder; PEP-3: Psychoeducational Profile Revised; R: Visual Reception (nonverbal problem solving); RBS-R: Repetitive Behaviours Scale Revised; RL/EL: Receptive Language/Expressive Language; RME: Reading the mind in the eyes test; RSPM: Raven’s Standard Progressive Matrices; SBIQ: Stanford Binet Intelligence Scales: 5th Edition; SCQ: Social Communication Questionnaire; SIPT-BMC: Bilateral Motor Coordination subtest of the Sensory Integration and Praxis Test; SQ: Systemizing Questionnaire, SRS-2: Social Responsiveness Scale; SSIS-RS: Social Skills Improvement System-Rating Scales, SSIS-SS: Social skills improvement system – Social skills scale; TAS-20: Toronto Alexithymia Scale; TAS20: Toronto Alexithymia Scale; TEQ: Toronto Empathy Questionnaire; ToM: Theory of Mind; TONI-4: Test of Non-verbal Intelligence 4; VABS-2: Vineland Adaptive Behaviour Scales; VISK: the Dutch version of the Children’s Social Behaviour Questionnaire; WAIS-3: Wechsler Adult Intelligence Scale: 3; WAIS-R: Wechsler Adult Intelligence Scale Revised; WASI: Wechsler Abbreviated Scale of Intelligence; WISC: Wechsler Intelligence Scale for Children; WPPSI: Wechsler Preschool and Primary Scale of Intelligence; WST: Wortschatztest (German verbal IQ test).

Of the 22 studies including a comparison group, 19 examined interactions between two human partners. Four of these involved participants and partners who were both adults and 15 involved child or infant participants partnered with adults (*n* = 14) or with other child participants (*n* = 1). Most studies examined SMS in unfamiliar partnerships: all four studies involving adult participants and 10 of the 15 studies involving child participants did so. Four of the remaining studies with children examined SMS between child/infant participants and their parents or caregivers (Fitzpatrick et al., 2016; Liu et al., 2022; Marsh et al., 2013; Yirmiya et al., 2006). An additional study compared SMS in familiar partnerships (an autistic child and their parent/caregiver) compared with SMS is unfamiliar partnerships (an autistic child and a research assistant) (Zampella et al., 2020). The seven studies that lacked a comparison group all involved human dyads, one including interactions between peers and drama facilitators (Ward et al., 2018), and three including child and therapist dyads measuring SMS over time, meaning partners’ familiarity likely developed over the course of the study (Dvir et al., 2020; Manders et al., 2021; Venuti et al., 2017). Two examined SMS in unfamiliar pairs, involving autistic child participants and study confederates or clinicians (Koehne et al., 2016; Romero et al., 2018). The final study involving human dyads was in two parts, the first involving unfamiliar child and experimenter dyads and the second involving children and music therapists over the course of an intervention, who also would have had the opportunity to grow in familiarity (Yoo & Kim, 2018). Therefore, 17 studies involving human partners measured SMS in unfamiliar pairs and 12 in familiar pairs.

#### Methods of measurement and analysis

Across 29 papers, this review identified 24 different tasks designed to elicit and measure synchrony, summarised in Table 2. Thirteen used experimental methods to assess both spontaneous synchrony, which occurs during dynamic interaction, and intentional synchrony, which requires conscious coordination with a partner, such as pendulum swinging (e.g., Fitzpatrick et al., 2016). Sixteen used naturalistic tasks, such as conversation activities, to assess only spontaneous synchrony (e.g., Georgescu et al., 2020), of which five were intervention studies, such as Dance and Movement Therapy (e.g., Koehne et al., 2016).

All papers used quantitative methods to measure synchrony and two papers also used qualitative descriptions of case studies (Manders et al., 2021; Ward et al., 2018). The majority (*n* = 21) used automated methods to measure synchrony, such as relative phase analysis from motion detectors (e.g., Marsh et al., 2013) or frame differencing methods using video-recordings, such as Motion Energy Analysis (e.g., Noel et al., 2017). One paper used a measure of co-confident (CC) motion, which parsed data into motion segments; the motion segments were considered CC, or synchronous, should they reach only one peak velocity (Brezis et al., 2017). Four papers used coherence-timing measures such as reaction time coherence (e.g., Stoit et al., 2011).

Eight papers used observational or manual-coding methods to measure synchrony (Dvir et al., 2020; Griffioen et al., 2020; Kaur et al., 2018; Koehne et al., 2016; Manders et al., 2021; Venuti et al., 2017; Zadok et al., 2022; Zampella et al., 2020). These methods included, for example, observational coding tools (Dvir et al., 2020; Venuti et al., 2017), manual coding of each partner’s behaviour, noting the start and finish of synchronous movements (Griffioen et al., 2020; Kaur et al., 2018), and behaviour ratings from naïve coders (Koehne et al., 2016; Manders et al., 2021; Zadok et al., 2022; Zampella et al., 2020).

### Data synthesis

This review has identified several types of evidence with relevance to SMS in autism, including quantitative, qualitative and intervention-based studies. The quantitative studies use several analysis techniques and distinct tasks to measure SMS, which does not permit ready quantitative comparison. We therefore qualitatively synthesise the data to address our primary aim, addressing whether current literature shows SMS differences in interactions involving an autistic partner (autistic pairs and non-autistic pairs) compared to mixed-neurotype dyads.

Of the 29 papers we identified, 19 included a comparison group and examined interactions between human partners. Fifteen of these indicated lower SMS in pairs involving an autistic person (i.e. mixed pairs or autistic pairs) compared with non-autistic pairs, but only two involved autistic pairs. This includes one paper which examined SMS in parents and infants with autistic siblings (SIBS-ASC) compared with parents and infants with non-autistic siblings (SIBS-NT). Differences were present across several tasks measuring spontaneous and intentional synchrony. In intentional tasks, autistic participants with a partner showed less synchrony than non-autistic participants did with their partner. Differences were present in joint balancing tasks (Stoit et al., 2011), object tapping or drumming (Kawasaki et al., 2017; Romero et al., 2016), a whole body-sway task (Su et al., 2021), and in two movement batteries: the Social Motor Battery (Fitzpatrick et al., 2017b; Romero et al., 2016) and the Motor Coordination Experimental Paradigm (Kaur et al., 2018). In spontaneous tasks, autistic participants with a non-autistic partner also showed lower synchrony than non-autistic participants did with their non-autistic partner. This included some spontaneous tasks that were mechanical in nature, such as interpersonal handclapping (Fitzpatrick et al., 2017b; Romero et al., 2016), puzzle-making (Delaherche et al., 2013), or joint improvisation (Brezis et al., 2017). However, other spontaneous tasks reflected naturalistic scenarios, including conversation tasks (Georgescu et al., 2020; Noel et al., 2017; Zadok et al., 2022; Zampella et al., 2020), diagnostic interviews (Koehler et al., 2022), or reading (Liu et al., 2022). Parents and infants of 14 months with SIBS-ASC also showed lower SMS with one another than parents and infants of 14 months with SIBS-NT did (Yirmiya et al., 2006).

Four of the 18 papers involving human partners show the common pattern of lower SMS in mixed-neurotype pairs compared with non-autistic pairs, but in each case, these differences did not appear in certain specific conditions. Fitzpatrick et al. (2013) found lower SMS in mixed pairs compared to non-autistic pairs in the SMS task battery, where participants were asked to follow an experimenter’s movement in a series of action sequences. However, similar SMS was observed between groups in one condition, the object-object condition, which involved following the experimenter’s movements of an object. When participants were asked to swing pendulums alongside a partner in Fitzpatrick et al.’s (2016) study, lower SMS was observed in mixed compared to non-autistic pairs in both the intentional and the spontaneous conditions when participants were asked to look at their partner, but there were no differences in SMS when partners were free to swing their pendulum while looking away from their partner. Marsh et al. (2013) found significantly less in-phase SMS patterns in mixed-neurotype pairs compared with non-autistic pairs when participants rocked in rocking chairs. However, there was no difference between groups for anti-phase SMS patterns. Finally, during reaching and grasping tasks, Fulceri et al. (2018) found lower SMS in mixed pairs compared with non-autistic pairs when the end-point of the task (i.e. their final reach point) was unclear. When the end-point of the task was clear, there were no significant group differences.

In three studies, participants interacted with virtual avatars. Autistic participants showed lower SMS than non-autistic participants did during intentional tasks but showed similarity in spontaneous tasks. Specifically, autistic children showed lower SMS than non-autistic children and children with DCD did when intentionally synchronising to an avatar that took the form of a tightrope walker, whose motion was based on human movements (Xavier et al., 2018). During a synchronous tapping task, autistic adults showed less SMS than non-autistic adults when intentionally synchronising to computer-generated sounds (Kawasaki et al., 2017). Kostrubiec et al.’s (2018) virtual coordination task required synchronising own movements of an on-screen dot with the movements of a virtual oscillating dot, also based on modelling of human motion. Autistic children displayed lower synchrony with the dot than non-autistic children did when asked to intentionally synchronise (Kostrubiec et al., 2018). However, there were no differences in SMS between autistic and non-autistic children when they were free to move their dot at their own pace (Kostrubiec et al., 2018).

The remaining seven papers included in this review did not include a comparison group. One described patterns of close SMS between a pair of autistic peers and between autistic children and facilitators (Ward et al., 2018). Six examined SMS between autistic children and non-autistic clinician partners or therapy dogs over time following a rhythm-mediated intervention, including Dance and Movement Therapy or Music Therapy (Dvir et al., 2020; Griffioen et al., 2020; Koehne et al., 2016; Manders et al., 2021; Venuti et al., 2017; Yoo & Kim, 2018). Five of the seven intervention studies found an increase in SMS from the first to the final session (Dvir et al., 2020; Griffioen et al., 2020; Koehne et al., 2016; Venuti et al., 2017; Yoo & Kim, 2018). In each of these five studies, participant and therapist dyads remained consistent over the course of the therapy sessions. Manders et al. (2021) found an increase in SMS from the first to the last session for one specific dyad, who were partnered together throughout the series of therapy sessions. However, the remaining four participants’ partners differed for each therapy session. For participants with inconsistent partnering, there was no significant change in SMS over the course of the therapy sessions (Manders et al., 2021). One of the six intervention studies and a final study that did not include a comparison group examined SMS compared with chance. They revealed SMS between autistic children and a clinician or therapy dog at levels higher than chance (Griffioen et al., 2020; Romero et al., 2018).

Only three of the 29 studies we identified included pairs with partners who were both autistic and only one of these directly compared all dyad types (autistic, non-autistic and mixed-neurotype pairs). Pairs of autistic children displayed lower SMS than non-autistic pairs when working cooperatively to raise a virtual balancing bar (Stoit et al., 2011). During conversation between two adult participants, mixed dyads and autistic dyads demonstrated comparable synchrony; however this was lower than non-autistic dyads (Georgescu et al., 2020). A final study in this set examined SMS in autistic and non-autistic pairs in a group drama performance (Ward et al., 2018). While comparisons were not drawn across groups, Ward et al. (2018) described instances of SMS between a pair of autistic peers and between the autistic children and non-autistic facilitators. While the studies involving autistic pairs in this review were limited to three of the 29 papers, the two comparative papers we found suggested lower SMS in dyads involving an autistic partner compared with non-autistic dyads.

## Discussion

This is the first study to systematically review the literature examining SMS in autism. Twenty-nine papers met our inclusion criteria. Of the 21 studies that included a comparison group, all indicated weaker SMS in interactions involving autistic partners (i.e., with another autistic, a non-autistic, or virtual partner) compared to interactions between two non-autistic people, or between a non-autistic participant and a virtual partner. Five of these reported mixed findings, and provide some evidence that autistic people and their partners can closely synchronise under certain conditions. These results point to some limitations in SMS research, which may have influenced the SMS displayed by autistic people and their partners in research so far. The remaining eight studies did not include a comparison group, instead examining SMS in mixed-neurotype pairs over time, in relation to chance or using qualitative descriptions of SMS patterns. Three of these provide evidence of close SMS in autistic people and their partners, with two indicating levels higher than chance. The five intervention studies all suggest SMS can increase over time in mixed-neurotype pairs following rhythmic interpersonal interventions, such as Improvisional Music Therapy (IMT). We note the current SMS literature is limited by the scarcity of research examining SMS in pairs of autistic participants. Only three papers identified involved autistic dyads, and only one of these investigated SMS between all three dyad types (mixed-neurotype pairs, autistic pairs and non-autistic pairs).

The findings from this review show that current SMS literature suggests weaker synchrony occurs in interactions when one or both partners are autistic, compared with interactions between two non-autistic people. The results appear mostly consistent for tasks tapping both intentional and spontaneous synchrony and across different methods to detect SMS, such as automated measurement, manual coding, or global judgement ratings. Fitzpatrick et al. (2016) proposed an SMS model of autism, where difficulties with SMS may underpin the social difficulties associated with autism. The literature so far appears to support this premise, and this review demonstrates that autistic people and their partners display weaker SMS than non-autistic pairs in the conditions studied so far.

However, we know that synchrony is an interpersonal measure. The Double Empathy Problem (Milton, 2012) and other theories associated with Interactional Heterogeneity (for a summary, see Georgescu et al., 2020) suggest partners of different neurotypes might both find it challenging to adapt their behaviour to their partner’s communication styles. It is therefore inconsistent to attribute an SMS deficit only to the autistic partner in mixed-neurotype interactions. We found few studies that included partners who were both autistic, which demonstrates the need for further studies of such pairs.

A better understanding of synchrony involves assessing whether partners equally adapt their movements in order to synchronise with their partner, or whether one partner consistently adapts their movements more by following their partner’s lead to facilitate synchrony. It is possible to determine each partners’ degree of following in only three out of the 26 studies involving human partners in the current review. Brezis et al. (2017) found autistic partners adapted their movements to their partner’s movements less than non-autistic partners did when explicitly instructed to do so. Marsh et al. (2013) also found lower SMS in autistic participants and their parents compared with non-autistic participants and their parents did when the parents’ movements were held constant (e.g., by rocking in time to a metronome), indicating less adaptation in autistic compared with non-autistic participants. Dvir et al. (2020) demonstrated that therapists increasingly followed a child’s movements over time, which facilitated an increase in SMS. However, the degree to which the child followed the therapists’ movements did not change over time. While the studies involving virtual partners involved movements based on human motion, they would not have the capacity to flexibly adapt their movements to the participants’ movements. This means it is also possible to establish the degree to which autistic and non-autistic participants adapt their movements to the movements of a virtual agent. In the three studies involving virtual partners, autistic participants showed weaker SMS compared with non-autistic people, indicating a lower tendency for autistic participants to adapt their movements to a virtual partner’s movements. While few studies examine the degree of leading and following in interactions involving an autistic partner, it is possible to identify some evidence to suggest that autistic people might be less likely to adapt their movements to facilitate synchrony with a partner compared with non-autistic people.

Six studies investigated SMS in autistic participants with therapists over a series of movement or music-based therapy sessions. While they did not include comparison groups, they demonstrate the importance of considering the relationship between interaction partners over time when examining SMS in autism. Approaches such as Dance and Movement Therapy place central importance on the developing relationship between the therapist and the client (Karkou & Sanderson, 2001). All six papers demonstrated close SMS between autistic children and their partners in therapeutic interactions and found an increase over time for pairs that remained consistent (Dvir et al., 2020; Griffioen et al., 2020; Koehne et al., 2016; Manders et al., 2021; Venuti et al., 2017; Yoo & Kim, 2018). Autistic people may need more time than other groups to develop rapport and trust upon meeting a new person (Robledo & Donnellan, 2016; Vivanti et al., 2018). Dvir et al. (2020) showed therapists attuned more to the children over a series of therapy sessions, indicating that it can take time even for NT partners trained in facilitating attuned interactions to spontaneously synchronise to an autistic child. Tickle-Degnen and Rosenthal (2009) also suggest synchrony becomes more important as a relationship grows. Synchrony between autistic people and unfamiliar partners may therefore take longer to appear. However, most studies in this review investigate synchrony in unfamiliar partnerships during one isolated interaction.

We identified some mixed results that reveal potential methodological limitations in SMS research. Principally, the task contexts and content may have influenced the degree of SMS observed in autistic people and their partners so far. First, there is some evidence that spontaneous synchrony might be easier to achieve for autistic people than intentional synchrony. One study found no differences between autistic and non-autistic children with virtual partners when they were free to spontaneously synchronise their movements (Kostrubiec et al., 2018) Another found differences in SMS between mixed-neurotype pairs and autistic pairs when they were asked to swing a pendulum in time with their partner and when they were free to move their pendulum while looking at their partner. However, there were no differences between groups when participants were asked to look away from their partner and were free to swing their pendulum at their own pace (Fitzpatrick et al., 2016). Other studies emphasise the need for observational methods that capture the of complexity of synchrony patterns. Marsh et al. (2013) found lower SMS in mixed-neurotype pairs compared with non-autistic pairs only for in-phase SMS patterns, there was no difference when comparing anti-phase patterns. Ward et al. (2018) found evidence of close SMS between autistic peers and between the autistic children and drama facilitators during when pairs did not appear socially engaged, such as close coupling of a child’s hand tapping to an adult’s global body movements. Task analyses show that intentional and spontaneous synchrony are discrete and load onto separate factors, suggesting they involve distinct processes (Fitzpatrick, 2018). Together these results underscore the need for further SMS research with autistic participants in natural and free-flowing interactions, which allow for the observation of multi-model and fine-grained SMS.

Intentional tasks generally involve additional processing demands, such as attention, working memory and movement planning, which would challenge autistic participants, who may have executive function difficulties (Craig et al., 2016). Autistic children are also known to have difficulties with praxis and movement planning, particularly for meaningless gestures, such as copying an experimenter’s movements (Gizzonio et al., 2015; Salowitz et al., 2013).

This could explain why differences in SMS are consistently found in intentional tasks but are sometimes not apparent in spontaneous tasks. For instance, interpersonal drumming and hand clapping require attention and working memory, and handclapping games, such as ‘pat-a-cake’, involve movement planning, where a child plans the steps to smoothly execute the sequence in an unfamiliar routine (Fitzpatrick et al., 2017b; Yoo & Kim, 2018). Other tasks require predicting a partner’s movements with no a priori information, which may be a challenge for autistic participants who can have difficulties with predictive processing (Palmer et al., 2015). This is illustrated by Fulceri et al. (2018), who compared cooperative reaching with a clear end-point (a single specified location) and with an unclear end-point (two possible locations). When the end-point was unclear, mixed pairs displayed lower synchrony than non-autistic pairs. However, SMS was comparable between dyads when the end-point was clearly indicated. In addition, Fitzpatrick et al. (2013) found mixed-neurotype pairs and autistic pairs only differed in SMS during the during the most complex and cognitively demanding condition, where participants were required to synchronise their movements to the experimenter’s movements while manipulating an object. There were no differences between groups in the four other conditions where participants were required to synchronise only to experimenters’ movements. Some spontaneous tasks also involve additional cognitive demands. Building a 3D puzzle requires working memory and attention skills, for example (Delaherche et al., 2013). These findings suggest additional cognitive demands present in some synchrony tasks may selectively disadvantage autistic participants and result in lower synchrony in autistic people and their partners compared with non-autistic pairs. They also stress the importance of task analysis when assessing SMS.

Understandably, studies of synchrony have typically used constrained and standardised tasks as we have described, which support precise control and replication. However, this may selectively disadvantage autistic participants and lab-based tasks can be arduous and lengthy, likely challenging for autistic people if the tasks are not adapted to their interests or meaningfully tailored. Williams (2020) demonstrated that supporting meaningful interactions between autistic participants in research, such as discussing common experiences of loneliness, could foster feelings of connectedness, which might support SMS. We identified one study that found close synchrony between autistic children and clinicians when the child chose the conversational topic (Romero et al., 2018). In contrast, conversation tasks where participants are given set topics revealed lower synchrony in autistic people and both autistic and non-autistic partners compared with pairs of non-autistic participants (Georgescu et al., 2020; Zampella et al., 2020). Tailoring tasks to participants’ interests and ensuring tasks are meaningful to autistic participants may encourage engagement (Murray et al., 2005), which could increase the likelihood of synchrony with a partner. Future work could consider differences in SMS in autistic people and their partners according to task design in greater depth.

Some autistic people need more time to habituate to new circumstances than non-autistic people do (Vivanti et al., 2018), and we know SMS is associated with social connectedness, which may take time to develop (Hove & Risen, 2009; Miles et al., 2009). Despite this, of the 21 studies including a comparison group, only two conducted SMS tasks in familiar environments and only five examined SMS in familiar partnerships: between children and their parents/caregivers. Parent-child partnerships would presumably have an established rapport and therefore display close SMS. Even so, five studies identified by this review found lower synchrony for parents and autistic children, or infants with autistic siblings compared to parents and non-autistic children or infants with non-autistic siblings (Fitzpatrick et al., 2016; Liu et al., 2022; Marsh et al., 2013; Yirmiya et al., 2006; Zampella et al., 2020). There was also no difference in SMS between autistic children and their parents, compared with the same child and a researcher (Zampella et al., 2020). We mentioned that it can take time for even therapists trained in facilitating attuned interactions to synchronise to an autistic child. Some research also suggests parent-child communication was more synchronous and reciprocal after parents received training to adapt their communication to their autistic child’s (Aldred et al., 2004). One study in this review found that when infants spontaneously acted as the ‘leader’ in a free-play interaction with their mother, mothers with infants who had autistic siblings followed their infant’s movements less than mothers and infants with NT siblings did (Yirmiya et al., 2006). These results demonstrate the need to examine SMS in a more nuanced way, by assessing whether partners equally adapt their movements in order to synchronise with their partner, or whether one partner more frequently adapts their movements to facilitate synchrony.

### Sample limitations

Most studies in this review involved autistic children (*n* = 23) or infants with autistic siblings (*n* = 1), rather than autistic adults (*n* = 5). Kostrubiec et al. (2018) found age correlated positively with social synchrony. If autistic people experience more synchrony in interactions as they get older, current results may underestimate the synchrony experienced by autistic adults and their partners. This may diminish the clinical utility of synchrony as a marker for adult diagnosis, which is becoming increasingly common and may be more complex than diagnosis during childhood (Huang et al., 2020). A pervasive issue in autism research is the heterogeneity of the population of people diagnosed as autistic. We have discussed how an interpersonal mismatch might make SMS challenging (Bolis et al., 2018). As relationships and social understanding appear closer between people with greater interpersonal similarity (Bolis et al., 2021; Crompton, Hallett, et al., 2020), involving familiar partners with similar levels of autistic traits, for example, will help our understanding of SMS in autistic people and their partners. Further, motor abilities between children and adults may differ simply according to their body size. Future work involving autistic peers could address these issues.

Many studies reviewed here cite their sample of autistic participants as ‘high-functioning’. This label is widely regarded as having little utility to describe the strengths and difficulties of autistic people but is used to describe autism in the absence of Intellectual Disability (ID; Hens et al., 2019). We found only one study involving minimally-verbal autistic children, which clearly describes some instances of successful social synchrony, owing to the flexible and inclusive methodology (Ward et al., 2018). However, the descriptive presentation of results does not permit quantitative comparison with other studies. There is therefore very little representation of autistic people who are minimally-verbal and/or with co-occurring ID, despite this group making up roughly 50% of the autistic population (Russell et al., 2019). This omission is unfortunate given the reported benefits and common use of client-led synchrony approaches including rhythm-mediated interventions and Intensive Interaction for this group (Delafield-Butt et al., 2020; Dvir et al., 2020; Griffioen et al., 2020). Some studies also explicitly exclude participants with co-occurring conditions, such as epilepsy and sensory processing differences (e.g., Xavier et al., 2018). This means only a subset, not fully representative of the autistic population, appears in current research (Lukmanji et al., 2019).

## Conclusion

The prevailing view in the literature has been that autistic people demonstrate impaired SMS. Fitzpatrick et al. (2016) posit that differences in SMS may underpin some social differences in autism. With this review, we aimed to determine whether the current literature shows consistent and robust SMS differences in pairs with one or more autistic partners compared with non-autistic pairs. Overall, results reveal lower SMS in interactions when one or both partners is autistic, compared with interactions between two non-autistic people. Few studies examine the degree of leading and following in SMS research, which makes it challenging to attribute an SMS deficit to one partner in an interaction. However, there is some evidence from the current review that autistic people are less likely than non-autistic people to adapt their movements to their partner’s movements to facilitate synchrony. Therefore, results of synchrony research so far seem to support an SMS model of autism, where SMS is weaker in interactions when one or more partners is autistic compared with non-autistic pairs.

However, some findings do not align with this pattern, and reveal indications that close synchrony may occur between autistic people and certain partners under certain conditions, which need further exploration. The differences in results for certain conditions potentially indicates methodological limitations in previous SMS research that can inform recommendations for future research. We found that naturalistic contexts allowing for free-flowing, spontaneous and meaningful interactions might be more likely than intentional, rhythmic tasks to capture synchrony between autistic people and their interactive partners. This may, in-part, be due to additional cognitive demands present in several SMS tasks, which could selectively disadvantage autistic participants. Synchrony between autistic people and their partners may also be heavily influenced by factors such as the environmental and social context. Specifically, familiarity of partner, novelty of the testing environment, and motivation to engage in the specific task will likely affect the performance of autistic participants more than non-autistic ones. Finally, very little research includes interactions between two autistic partners, which is problematic given the potential for closer synchrony to occur when partners are interpersonally similar. Our understanding of SMS in autism can be developed with research including pairs of autistic people within natural and familiar contexts using measurements that allow for identification of different modes of synchrony (e.g., Ward et al., 2018), and by closely tailoring SMS tasks to the needs and interests of autistic participants.

## Implications

This review shows that task analysis is important for understanding the sources of any differences in SMS found in interactions involving an autistic participant compared to those with non-autistic pairs. Understanding the social conditions where SMS is closest for autistic people and their partners would inform a clearer theoretical understanding of the role and development of synchrony in interaction with potential implications for interventions and approaches to working with autistic people. Therapeutic interactions between autistic clients and practitioners who are trained in facilitating attuned interactions clearly have potential to yield close SMS (e.g., Romero et al., 2018). Several papers reviewed indicate that SMS increases following person-centred and rhythmic interventions designed to build social connections and relationships (e.g., Dvir et al., 2020). Participation over time in familiar, client-led interactions that provide the opportunity for attunement to develop might therefore support early social interaction patterns. Given the importance of early social interaction for social and cognitive development, such approaches might then have continued benefits for social relationships in adulthood (Moll & Tomasello, 2007; Umberson and Karas Montez, 2010). Interventions that involve following an autistic partner’s movements or support synchronisation to an avatar modelled on human movement (e.g., Griffioen et al., 2020; Kostrubiec et al., 2018) might also increase the likelihood of mutual adaptation, which could generalise to other social contexts. At the very least, such approaches would allow autistic people to engage in experiences that support social and cognitive development and facilitate the development of closer social bonds.

## Supplemental Material

sj-docx-1-aut-10.1177_13623613231213295 – Supplemental material for Social motor synchrony in autism spectrum conditions: A systematic review Supplemental material, sj-docx-1-aut-10.1177_13623613231213295 for Social motor synchrony in autism spectrum conditions: A systematic review by Devyn Glass and Nicola Yuill in Autism

## References

[bibr1-13623613231213295] American Psychiatric Association. (2013). Diagnostic and statistical manual of mental disorders (5th ed.).

[bibr2-13623613231213295] Aldred C. Green J. Adams C . (2004). A new social communication intervention for children with autism: Pilot randomised controlled treatment study suggesting effectiveness. Journal of Child Psychology and Psychiatry and Allied Disciplines, 45(8), 1420–1430. 10.1111/j.1469-7610.2004.00338.x 15482502

[bibr3-13623613231213295] Baldwin M. Zhuoni Xiao M. Murray A. (2022). Temporal synchrony in autism: A systematic review. Review Journal of Autism and Developmental Disorders, 9(4), 596–617. 10.1007/s40489-021-00276-5

[bibr4-13623613231213295] Bernieri F. J. Rosenthal R. (1991). Interpersonal coordination: Behavior matching and interactional synchrony. In Feldman R. S. Rimé B. (Eds.), Studies in emotion & social interaction. Fundamentals of nonverbal behavior (pp. 401–432). Cambridge University Press; Editions de la Maison des Sciences de l’Homme.

[bibr5-13623613231213295] Bolis D. Balsters J. Wenderoth N. Becchio C. Schilbach L. (2018). Beyond autism: Introducing the dialectical misattunement hypothesis and a Bayesian account of intersubjectivity. Psychopathology, 50(6), 355–372. 10.1159/000484353 29232684

[bibr6-13623613231213295] Bolis D. Lahnakoski J. L. Seidel D. Tamm J. Schilbach L. (2021). Interpersonal similarity of autistic traits predicts friendship quality. Social Cognitive and Affective Neuroscience, 16(1–2), 222–231. 10.1093/scan/nsaa147 33104781 PMC7812635

[bibr7-13623613231213295] Bowsher-Murray C. Gerson S. Von Dem Hagen E. Jones C. R. G. (2022). The components of interpersonal synchrony in the typical population and in autism: A conceptual analysis. Frontiers in Psychology, 13, Article 897015. 10.3389/fpsyg.2022.897015 PMC9208202 35734455

[bibr8-13623613231213295] Brezis R. S. Noy L. Alony T. Gotlieb R. Cohen R. Golland Y. Levit-Binnun N. (2017). Patterns of joint improvisation in adults with autism spectrum disorder. Frontiers in Psychology, 8, 1–18. 10.3389/fpsyg.2017.01790 29114236 PMC5660713

[bibr9-13623613231213295] Charman T. (2011). Commentary: Glass half full or half empty? Testing social communication interventions for young children with autism – Reflections on Landa, Holman, O’Neill, and Stuart (2011). Journal of Child Psychology and Psychiatry and Allied Disciplines, 52(1), 22–23. 10.1111/j.1469-7610.2010.02359.x 21143228

[bibr10-13623613231213295] Chartrand T. L. Lakin J. L. (2012). The antecedents and consequences of human behavioral mimicry. Annual Review of Psychology, 64, 285–308. 10.1146/annurev-psych-113011-143754 23020640

[bibr11-13623613231213295] Chevallier C. Kohls G. Troiani V. Brodkin E. S. Schultz R. T. (2012). The social motivation theory of autism. Trends in Cognitive Science, 16(4), 231–239. 10.1016/j.tics.2012.02.007 PMC3329932 22425667

[bibr12-13623613231213295] Collignon O. Charbonneau G. Peters F. Nassim M. Lassonde M. Lepore F. Mottron L. Bertone A. (2013). Reduced multisensory facilitation in persons with autism. Cortex, 49(6), 1704–1710. 10.1016/j.cortex.2012.06.001 22818902

[bibr13-13623613231213295] Condon W. S. Sander L. W. (1974). Neonate movement is synchronized with adult speech: Interactional participation and language acquisition. Science, 183, 99–101. 4808791 10.1126/science.183.4120.99

[bibr14-13623613231213295] Craig F. Margari F. Legrottaglie A. R. Palumbi R. De Giambattista C. Margari L. (2016). A review of executive function deficits in autism spectrum disorder and attention-deficit/ hyperactivity disorder. Neuropsychiatric Disease and Treatment, 12, 1191–1202. 10.2147/NDT.S104620 PMC4869784 27274255

[bibr15-13623613231213295] Crompton C. J. Hallett S. Ropar D. Flynn E. Fletcher-Watson S. (2020). ‘I never realised everybody felt as happy as I do when I am around autistic people’: A thematic analysis of autistic adults’ relationships with autistic and neurotypical friends and family. Autism, 24(6), 1438–1448. 10.1177/1362361320908976 PMC7376620 32148068

[bibr16-13623613231213295] Crompton C. J. Ropar D. vans-Williams C. V. M. Flynn E. G. Fletcher-Watson S. (2020). Autistic peer to peer information transfer is highly effective. Preprint, 44, 1–19. 10.31219/OSF.IO/J4KNX PMC7545656 32431157

[bibr17-13623613231213295] De Jaegher H. Pieper B. Clénin D. Fuchs T . (2017). Grasping intersubjectivity: An invitation to embody social interaction research. Phenomenology and the Cognitive Sciences, 16(3), 491–523. 10.1007/s11097-016-9469-8

[bibr18-13623613231213295] Delafield-Butt J. T. Zeedyk M. S. Harder S. Vaever M. S. Caldwell P . (2020). Making meaning together: embodied narratives in a case of severe autism. Psychopathology, 53(2), 60–73. 32422641 10.1159/000506648 PMC8619765

[bibr19-13623613231213295] Delaherche E. Chetouani M. Bigouret F. Xavier J. Plaza M. Cohen D. (2013). Assessment of the communicative and coordination skills of children with Autism Spectrum Disorders and typically developing children using social signal processing. Research in Autism Spectrum Disorders, 7, 741–756. 10.1016/j.rasd.2013.02.003

[bibr20-13623613231213295] Dvir T. Lotan N. Viderman R. Elefant C. (2020). The body communicates: Movement synchrony during music therapy with children diagnosed with ASD. Arts in Psychotherapy, 69, 1–9. 10.1016/j.aip.2020.101658

[bibr21-13623613231213295] Feldman R. (2007). Parent-infant synchrony and the construction of shared timing; physiological precursors, developmental outcomes, and risk conditions. Journal of Child Psychology and Psychiatry and Allied Disciplines, 48(3–4), 329–354. 10.1111/j.1469-7610.2006.01701.x 17355401

[bibr22-13623613231213295] Feldman R. Magori-Cohen R. Galili G. Singer M. Louzoun Y. (2011). Mother and infant coordinate heart rhythms through episodes of interaction synchrony. Infant Behavior and Development, 34, 569–577. 10.1016/j.infbeh.2011.06.008 21767879

[bibr23-13623613231213295] Fitzpatrick P. (2018). The future of autism research: Dynamic and process-oriented approaches. Journal of the American Academy of Child and Adolescent Psychiatry, 57(1), 16–17. 10.1016/j.jaac.2017.11.001 29301661

[bibr24-13623613231213295] Fitzpatrick P. Dioro R. Richardson M. J. Schmidt R. C. (2013). Dynamical methods for measuring the time-dependent unfolding of social coordination in children with autism. Frontiers in Psychology, 7(21), 1–13. 10.3389/fnint.2013.00021 PMC3619188 23580133

[bibr25-13623613231213295] Fitzpatrick P. Frazier J. A. Cochran D. M. Mitchell T. Coleman C. Schmidt R. C. (2016). Impairments of social motor synchrony evident in autism spectrum disorder. Frontiers in Psychology, 7, Article 1323. 10.3389/fpsyg.2016.01323 PMC5005316 27630599

[bibr26-13623613231213295] Fitzpatrick P. Romero V. Amaral J. L. Duncan A. Barnard H. Richardson M. J. Schmidt R. C. (2017a). Evaluating the importance of social motor synchronization and motor skill for understanding autism. Autism Research, 10(10), 1687–1699. 10.1002/aur.1808 28590041 PMC5648610

[bibr27-13623613231213295] Fitzpatrick P. Romero V. Amaral J. L. Duncan A. Barnard H. Richardson M. J. Schmidt R. C. (2017b). Social motor synchronization: Insights for understanding social behavior in autism. Journal of Autism and Developmental Disorders, 47(7), 2092–2107. 10.1007/s10803-017-3124-2 28425022

[bibr28-13623613231213295] Frith U. (1996). Cognitive explanations of autism. Acta Pædiatrica, 85(416), 63–68. 10.1111/J.1651-2227.1996.TB14280.X 8997451

[bibr29-13623613231213295] Fulceri F. Tonacci A. Lucaferro A. Apicella F. Narzisi A. Vincenti G. Muratori F. Contaldo A. (2018). Interpersonal motor coordination during joint actions in children with and without autism spectrum disorder: The role of motor information. Research in Developmental Disabilities, 80, 13–23. 10.1016/j.ridd.2018.05.018 29879613

[bibr30-13623613231213295] Georgescu A. L. Koeroglu S. de C. Hamilton A. F. Vogeley K. Falter-Wagner C. M. Tschacher W. (2020). Reduced nonverbal interpersonal synchrony in autism spectrum disorder independent of partner diagnosis: A motion energy study. Molecular Autism, 11(11), 1–14. 10.1186/s13229-019-0305-1 32014017 PMC6998161

[bibr31-13623613231213295] Gizzonio V. Avanzini P. Campi C. Orivoli S. Piccolo B. Cantalupo G. Tassinari C. A. Rizzolati G. Fabbri-Destro M . (2015). Failure in pantomime action execution correlates with the severity of social behavior deficits in children with autism: A praxis study. Journal of Autism and Developmental Disorders, 45, 3085–3097. 10.1007/s10803-015-2461-2 25962471

[bibr32-13623613231213295] Green J. Pickles A. Pasco G. Bedford R. Wan M. W. Elsabbagh M. Slonims V. Gliga T. Jones E. Cheung C. Charman T. Johnson M. (2017). Randomised trial of a parent-mediated intervention for infants at high risk for autism: Longitudinal outcomes to age 3 years. Journal of Child Psychology and Psychiatry, 58(12), 1330–1340. 10.1111/jcpp.12728 28393350 PMC5724485

[bibr33-13623613231213295] Griffioen R. E. Steen S. Verheggen T. Enders-Slegers M. Cox R. (2020). Changes in behavioural synchrony during dog-assisted therapy for children with autism spectrum disorder and children with Down syndrome. Journal of Applied Research in Intellectual Disabilities, 33(3), 398–408. 10.1111/jar.12682 31809563

[bibr34-13623613231213295] Grimes D. A. Schulz K. F. (2002). Bias and causal associations in observational research. Lancet, 359, 248–252. 10.1016/S0140-6736(02)07451-2 11812579

[bibr35-13623613231213295] Hens K. Robeyns I. Schaubroek K. (2019). The ethics of autism. Philosophy Compass, 14(1), 1–11.

[bibr36-13623613231213295] Hong Q. N. Pluye P. Fàbregues S. Bartlett G. Boardman F. Cargo M. Dagenais P. Gagnon M. P. Griffiths F. Nicolau B. O’Cathain A. Rousseau M. C. Vedel I. (2018). Mixed Methods Appraisal Tool (MMAT) (Version 2018). Registration of Copyright (#1148552), Canadian Intellectual Property Office, Industry Canada.

[bibr37-13623613231213295] Hove M. J. Risen J. L. (2009). It’s all in the timing: Interpersonal synchrony increases affiliation. Social Cognition, 27(6), 949–960. 10.1521/soco.2009.27.6.949

[bibr38-13623613231213295] Huang Y. Arnold S. R. C. Foley K. R. Trollor J. N. (2020, August 1). Diagnosis of autism in adulthood: A scoping review. Autism, 24, 1311–1327. 10.1177/1362361320903128 32106698

[bibr39-13623613231213295] Karkou V. Sanderson P. (2001). Dance movement therapy in the UK: A field emerging from dance education. European Physical Education Review, 7(2), 137–155. 10.1177/1356336X010072003

[bibr40-13623613231213295] Kaur M. Srinivasan S. M. Bhat A. N. (2018). Comparing motor performance, praxis, coordination, and interpersonal synchrony between children with and without Autism Spectrum Disorder (ASD). Research in Developmental Disabilities, 72, 79–95. 10.1016/j.ridd.2017.10.025 29121516 PMC5743591

[bibr41-13623613231213295] Kawasaki M. Kitajo K. Fukao K. Murai T. Yamaguchi Y. Funabiki Y. (2017). Frontal theta activation during motor synchronization in autism. Scientific Reports, 7, Article 15034. 10.1038/s41598-017-14508-4 PMC5678163 29118348

[bibr42-13623613231213295] Koehler J. C. Georgescu A. L. Weiske J. Spangemacher M. Burghof L. Falkai P. Falter-Wagner C. M. (2022). Brief report: Specificity of interpersonal synchrony deficits to autism spectrum disorder and its potential for digitally assisted diagnostics. Journal of Autism and Developmental Disorders, 52, 3718–3726. 10.1007/s10803-021-05194-3 34331629 PMC9296396

[bibr43-13623613231213295] Koehne S. Behrends A. Fairhurst M. T. Dziobek I. (2016). Fostering social cognition through an imitation- and synchronization-based dance/movement intervention in adults with autism spectrum disorder: A controlled proof-of-concept study. Psychotherapy and Psychosomatics, 85(1), 27–35. 10.1159/000441111 26609704

[bibr44-13623613231213295] Konvalinka I. Xygalatas D. Bulbulia J. Schjødt U. Jegindø E. M. Wallot S. Roepstorff A. (2011). Synchronized arousal between performers and related spectators in a fire-walking ritual. Proceedings of the National Academy of Sciences of the United States of America, 108(20), 8514–8519. 10.1073/pnas.1016955108 21536887 PMC3100954

[bibr45-13623613231213295] Kostrubiec V. Huys R. Jas B. Kruck J. (2018). Age-dependent relationship between socio-adaptability and motor coordination in high functioning children with autism spectrum disorder. Journal of Autism and Developmental Disorders, 48(1), 209–224. 10.1007/s10803-017-3326-7 28975439

[bibr46-13623613231213295] Liu T. Schultz B. G. Dai D. Liu C. Lense M. D. (2022). Parent-child nonverbal engagement during read versus sung book-sharing in preschoolers with and without ASD. Psychology of Music, 50, 1721–1739. 10.1177/03057356211058781 36381385 PMC9648075

[bibr47-13623613231213295] Loth E. Ahmad J. Chatham C. López B. Carter B. Crawley D. Oakley B. Hayward H. Cooke J. Cáceres A. S. J. Bzdok D. Jones E. Charman T. Beckmann C. Bourgeron T. Toro R. Buitelaar J. Murphy D. Dumas G. (2021). The meaning of significant mean group differences for biomarker discovery. PLOS Computational Biology, 17(11), 1–16. 10.1371/journal.pcbi.1009477 PMC8601419 34793435

[bibr48-13623613231213295] Lukmanji S. Manji S. A. Kadhim S. Sauro K. M. Wirrell E. C. Kwon C. S. Jetté N. (2019). The co-occurrence of epilepsy and autism: A systematic review. Epilepsy and Behavior, 98, 238–248. 10.1016/j.yebeh.2019.07.037 31398688

[bibr49-13623613231213295] Manders E. Goodill S. Koch S. C. Giarelli E. Polansky M. Fisher K. Fuchs T. (2021). The mirroring dance: Synchrony and interaction quality of five adolescents and adults on the autism spectrum in dance/movement therapy. Frontiers in Psychology, 12, Article 717389. 10.3389/FPSYG.2021.717389/FULL PMC8551749 34721165

[bibr50-13623613231213295] Marsh K. L. Isenhower R. W. Richardson M. J. Helt M. Verbalis A. D. Schmidt R. C. Fein D. (2013). Autism and social disconnection in interpersonal rocking. Frontiers in Integrative Neuroscience, 7, 1–8. 10.3389/fnint.2013.00004 23423608 PMC3575023

[bibr51-13623613231213295] Mcnaughton K. A. Redcay E. (2020). Interpersonal synchrony in autism. Autism Spectrum Disorders, 22(12), 1–11. 10.1007/s11920-020-1135-8 32025922

[bibr52-13623613231213295] Miles L. K. Nind L. K. Macrae C. N. (2009). The rhythm of rapport: Interpersonal synchrony and social perception. Journal of Experimental Social Psychology, 45, 585–589. 10.1016/j.jesp.2009.02.002

[bibr53-13623613231213295] Milton D. E. M. (2012). On the ontological status of autism: The ‘double empathy problem’. Disability and Society, 27(6), 883–887. 10.1080/09687599.2012.710008

[bibr54-13623613231213295] Moll H. Tomasello M. (2007). Cooperation and human cognition: The Vygotskian intelligence hypothesis. Philosophical Transactions of the Royal Society B: Biological Sciences, 362(1480), 639–648. 10.1098/rstb.2006.2000 PMC2346522 17296598

[bibr55-13623613231213295] Murray D. Lesser M. Lawson W. (2005). Attention, monotropism and the diagnostic criteria for autism. Autism, 9(2), 139–156. 10.1177/1362361305051398 15857859

[bibr56-13623613231213295] Noel J. P. De Niear M. Lazzara N. S. Wallace M. T. (2017). Uncoupling between multisensory temporal function and non-verbal turn-taking in autism spectrum disorder. IEEE Transactions on Cognitive and Developmental Systems, 10(4), 973–982. 10.1109/TCDS.2017.2778141

[bibr57-13623613231213295] Ouzzani M. Hammady H. Fedorowicz Z. Elmagarmid A. (2016). Rayyan – A web and mobile app for systematic reviews. Systematic Reviews, 5(210), 1–10. 10.1186/s13643-016-0384-4 27919275 PMC5139140

[bibr58-13623613231213295] Page M. J. McKenzie J. E. Bossuyt P. M. Boutron I. Hoffmann T. C. Mulrow C. D. Moher D. (2021, March 29). The PRISMA 2020 statement: An updated guideline for reporting systematic reviews. The BMJ, 372, n71. 10.1136/bmj.n71 PMC8005924 33782057

[bibr59-13623613231213295] Palmer C. J. Seth A. K. Hohwy J. (2015). The felt presence of other minds: Predictive processing, counterfactual predictions, and mentalising in autism. Consciousness and Cognition, 36, 376–389. 25934216 10.1016/j.concog.2015.04.007

[bibr60-13623613231213295] Pennington M. L. Cullinan D. Southern L. B. (2014). Defining autism: Variability in state education agency definitions of and evaluations for autism spectrum disorders. Autism Research and Treatment, 2014, 1–8. 10.1155/2014/327271 PMC4060325 24987527

[bibr61-13623613231213295] Prepin K. Pelachaud C. (2013). Basics of intersubjectivity dynamics: Model of synchrony emergence when dialogue partners understand each other. Communications in Computer and Information Science, 271, 302–318. 10.1007/978-3-642-29966-7_20

[bibr62-13623613231213295] Rabinowitch T. C. Knafo-Noam A. (2015). Synchronous rhythmic interaction enhances children’s perceived similarity and closeness towards each other. PLOS ONE, 10(4), 1–10. 10.1371/journal.pone.0120878 PMC4390221 25853859

[bibr63-13623613231213295] Ramseyer F. Tschacher W. (2011). Nonverbal synchrony in psychotherapy: Coordinated body movement reflects relationship quality and outcome. Journal of Consulting and Clinical Psychology, 79(3), 284–295. 10.1037/a0023419 21639608

[bibr64-13623613231213295] Robledo J. Donnellan A. M. (2016). Supportive relationships in autism spectrum disorder: Perspectives of individuals with ASD and supporters. Behavioral Sciences, 6(23), 1–50. 10.3390/bs6040023 PMC5197936 27827873

[bibr65-13623613231213295] Romero V. Fitzpatrick P. Roulier S. Duncan A. Richardson M. J. Schmidt R. C. (2018). Evidence of embodied social competence during conversation in high functioning children with autism spectrum disorder. PLOS ONE, 13(4), e0195888. 10.1371/journal.pone.0193906 PMC5837293 29505608

[bibr66-13623613231213295] Romero V. Fitzpatrick P. Schmidt R. C. Richardson M. J. (2016). Recurrence plots and their quantifications: Expanding horizons. Recurrence Plots and Their Quantifications: Expanding Horizons, 180(June), 17–19. 10.1007/978-3-319-29922-8

[bibr67-13623613231213295] Russell G. Kapp S. K. Elliott D. Elphick C. Gwernan-Jones R. Owens C. (2019). Mapping the autistic advantage from the accounts of adults diagnosed with autism: A qualitative study. Autism in Adulthood, 1(2), 124–133. 10.1089/aut.2018.0035 31058260 PMC6493410

[bibr68-13623613231213295] Salowitz N. M. G. Eccarius P. Karst J. Carson A. Schohl K. Stevens S. Vaughn Van Hecke A. Scheidt R. A. (2012). Brief report: Visuo-spatial guidance of movement during gesture imitation and mirror drawing in children with autism spectrum disorders. Journal of Autism and Developmental Disorder, 43, 985–995. 10.1007/s10803-012-1631-8 22898762

[bibr69-13623613231213295] Shamseer L. Moher D. Clarke M. Ghersi D. Liberati A. Petticrew M. Shekelle P. Stewart L. A. (2015). Preferred reporting items for systematic review and meta-analysis protocols (PRISMA-P) 2015: Elaboration and explanation. BMJ, 349, g7647. 10.1136/bmj.g7647 25555855

[bibr70-13623613231213295] Stoet G. Lopez B. (2011). Task-switching abilities in children with autism spectrum disorder. The European Journal of Developmental Psychology, 8(2), 244–260. 10.1080/17405629.2010.492000

[bibr71-13623613231213295] Stoit A. M. B. Van Schie H. T. Riem M. Meulenbroek R. G. J. Newman-Norlund R. D. Slaats-Willemse D. I. E. Buitelaar J. K. (2011). Internal model deficits impair joint action in children and adolescents with autism spectrum disorders. Research in Autism Spectrum Disorders, 5, 1526–1537. 10.1016/j.rasd.2011.02.016

[bibr72-13623613231213295] Su W. C. Culotta M. K. Tsuzuki D. Bhat A. (2021). Movement kinematics and cortical activation in children with and without autism spectrum disorder during sway synchrony tasks: An fNIRS study. Scientific Reports, 11(1), 15035. 10.1038/s41598-021-94519-4 34294815 PMC8298433

[bibr73-13623613231213295] Tickle-Degnen L. Rosenthal R. (2009). The nature of rapport and its nonverbal correlates. Psychological Inquiry, 1(4), 285–293. 10.1207/s15327965pli0104_1

[bibr74-13623613231213295] Tuncgenc B. Cohen E. (2016). Movement synchrony forges social bonds across group divides. Frontiers in Psychology, 8(5), 7837–7842. 10.3389/fpsyg.2016.00782 PMC4882973 27303341

[bibr75-13623613231213295] Umberson D. Karas Montez J. (2010). Social relationships and health: A flashpoint for health policy NIH public access. Journal of Health and Social Behavior, 51, 54–66. 10.1177/0022146510383501 PMC3150158 20943583

[bibr76-13623613231213295] Valdesolo P. Ouyang J. DeSteno D. (2010). The rhythm of joint action: Synchrony promotes cooperative ability. Journal of Experimental Social Psychology, 46(4), 693–695. 10.1016/j.jesp.2010.03.004

[bibr77-13623613231213295] Venuti P. Bentenuto A. Cainelli S. Landi I. Suvini F. Tancredi R. Muratori F. (2017). A joint behavioral and emotive analysis of synchrony in music therapy of children with autism spectrum disorders. Health Psychology Report, 5(2), 162–172. 10.5114/hpr.2017.63985

[bibr78-13623613231213295] Vivanti G. Hocking D. R. Fanning P. A. J. Uljarevic M. Postorino V. Mazzone L. Drexel A. J. (2018). Attention to novelty versus repetition: Contrasting habituation profiles in Autism and Williams syndrome. Developmental Cognitive Neuroscience, 29, 54–60. 10.1016/j.dcn.2017.01.006 28130077 PMC6987850

[bibr79-13623613231213295] Vygotsky L. S. (1978). Mind in society. The development of higher psychological processes. Harvard University Press.

[bibr80-13623613231213295] Ward J. A. Richardson D. Orgs G. Hunter K. Hamilton A. (2018, October). Sensing interpersonal synchrony between actors and autistic children in theatre using wrist-worn accelerometers. In 22nd International Symposium on Wearable Computers, ISWC (pp. 148–155). Association for Computing Machinery. 10.1145/3267242.3267263

[bibr81-13623613231213295] Williams G. (2020). From anonymous subject to engaged stakeholder: Enriching participant experience in autistic-language-use research. Research for All, 4(2), 314–328.

[bibr82-13623613231213295] Xavier J. Gauthier S. Cohen D. Zahoui M. Chetouani M. Villa F. Anzalone S. (2018). Interpersonal synchronization, motor coordination, and control are impaired during a dynamic imitation task in children with autism spectrum disorder. Frontiers in Psychology, 9, 1–10. 10.3389/fpsyg.2018.01467 30233439 PMC6129607

[bibr83-13623613231213295] Yirmiya N. Gamliel I. Pilowsky T. Feldman R. Baron-Cohen S. Sigman M. (2006). The development of siblings of children with autism at 4 and 14 months: Social engagement, communication, and cognition. Journal of Child Psychology and Psychiatry, 47(5), 511–523. 10.1111/j.1469-7610.2005.01528.x 16671934

[bibr84-13623613231213295] Yoo G. E. Kim S. J. (2018). Dyadic drum playing and social skills: Implications for rhythm-mediated intervention for children with autism spectrum disorder. Journal of Music Therapy, 55(3), 340–375. 10.1093/jmt/thy013 30137544

[bibr85-13623613231213295] Zadok E. Gordon I. Navon R. Rabin S. J. Golan O. (2022). Shifts in behavioral synchrony in response to an interaction partner’s distress in adolescents with and without ASD. Journal of Autism and Developmental Disorders, 52, 4261–4273. 10.1007/s10803-021-05307-y 34611838

[bibr86-13623613231213295] Zampella C. J. Csumitta K. D. Simon E. Bennetto L. (2020). Interactional synchrony and its association with social and communication ability in children with and without autism spectrum disorder. Journal of Autism and Developmental Disorders, 50, 3195–3206. 10.1007/s10803-020-04412-8 32065341 PMC7569722

