# Supplementary material for: Social motor synchrony in autism spectrum conditions: A systematic review
Source: Autism. 2023 Nov 28;28(7):1638–53. doi: 10.1177/13623613231213295 (PMC11193327; doi:10.1177/13623613231213295)
Supplement: sj-docx-1-aut-10.1177_13623613231213295 – Supplemental material for Social motor synchrony in autism spectrum conditions: A systematic review [file sj-docx-1-aut-10.1177_13623613231213295.docx]

# Supplementary Material

## Appendix 1: Search Strategies

### SCOPUS

| ASC search terms | (TITLE-ABS-KEY ( "Autism Spectrum Disorder*" OR "Autism Spectrum Condition*" OR autis* OR "ASD" OR "ASC" OR "Autis* Traits" OR "Autis* Characteristics" OR asperger* ) |
| --- | --- |
| SMS search terms | AND TITLE-ABS-KEY ( "Social Motor Sync*" OR "Interpersonal Sync*" OR sync* OR "Motor Sync*" OR "Interactional Sync*" OR "Social Sync*" OR "Behavioural Sync*" OR "Behavioral Sync*" OR "Temporal Sync*" OR "Rhythmic Movment" OR "Temporal Dynamics" OR "Coordinat*" OR "Interpersonal Coordinat*" OR "Social* Coordinat*" OR "Motor Coordination" OR "Bod* Coordination" OR "Rhythm*" OR “Interpersonal Coordinat*” OR “Social Coordinat*” OR “Motor Coordinat*” OR “Bod* Coordinat*”) ) |
| Limiters: subject area | AND ( EXCLUDE ( SUBJAREA , "BIOC" ) OR EXCLUDE ( SUBJAREA , "ENGI" ) OR EXCLUDE ( SUBJAREA , "AGRI" ) OR EXCLUDE ( SUBJAREA , "PHYS" ) OR EXCLUDE ( SUBJAREA , "MATH" ) OR EXCLUDE ( SUBJAREA , "PHAR" ) OR EXCLUDE ( SUBJAREA , "IMMU" ) OR EXCLUDE ( SUBJAREA , "CENG" ) OR EXCLUDE ( SUBJAREA , "CHEM" ) OR EXCLUDE ( SUBJAREA , "EART" ) OR EXCLUDE ( SUBJAREA , "ENVI" ) OR EXCLUDE ( SUBJAREA , "VETE" ) OR EXCLUDE ( SUBJAREA , "DENT" ) OR EXCLUDE ( SUBJAREA , "NEUR" ) OR EXCLUDE ( SUBJAREA , "MEDI" ) OR EXCLUDE ( SUBJAREA , "HEAL" ) OR EXCLUDE ( SUBJAREA , "NURS" ) OR EXCLUDE ( SUBJAREA , "MATE" ) ) OR EXCLUDE ( SUBJAREA , "ENER" ) ) |
| Limiters: publication type | AND (LIMIT-TO (DOCTYPE, "ar" ) OR LIMIT-TO ( DOCTYPE, "re" ) OR LIMIT-TO ( DOCTYPE , "cp" ) OR LIMIT-TO ( DOCTYPE, "ip" )) |
| Limiters: keywords | AND ( EXCLUDE ( EXACTKEYWORD , "Metabolism" ) OR EXCLUDE  ( EXACTKEYWORD , "Animals" ) OR EXCLUDE ( EXACTKEYWORD , "Electroencephalography" ) OR EXCLUDE ( EXACTKEYWORD , "Excitatory Postsynaptic Potential" ) OR EXCLUDE ( EXACTKEYWORD , "Mice" ) OR EXCLUDE ( EXACTKEYWORD , "Mouse" ) OR EXCLUDE ( EXACTKEYWORD , "Nonhuman" ) OR EXCLUDE ( EXACTKEYWORD , "Action Potential" ) OR EXCLUDE ( EXACTKEYWORD , "Action Potentials" ) OR EXCLUDE ( EXACTKEYWORD , "Nerve Cell Network" ) OR EXCLUDE ( EXACTKEYWORD , "Nerve Net" ) OR EXCLUDE ( EXACTKEYWORD , "Sleep Disorder" ) OR EXCLUDE ( EXACTKEYWORD , "Photic Stimulation" ) OR EXCLUDE ( EXACTKEYWORD , "Sleep" ) OR EXCLUDE ( EXACTKEYWORD , "Phenotype" ) OR EXCLUDE ( EXACTKEYWORD , "Sleep Disorders" ) OR EXCLUDE ( EXACTKEYWORD , "Depression" ) OR EXCLUDE ( EXACTKEYWORD , "Hydrocortisone" ) OR EXCLUDE ( EXACTKEYWORD , "Photostimulation" ) ) |

### Web of Science

| ASC search terms | TOPIC: ("Autism Spectrum Disorder*" OR "Autism Spectrum Condition*" OR autis* OR "ASD" OR "ASC" OR "Autis* Traits" OR "Autis* Characteristics" OR Asperger*) |
| --- | --- |
| SMS search terms | AND TOPIC: ("Social Motor Sync*" OR "Interpersonal Sync*" OR sync* OR "Motor Sync*" OR "Interactional Sync*" OR "Social Sync*" OR "Behavioural Sync*" OR "Behavioral Sync*" OR "Temporal Sync*" OR “Rhythmic Movement" OR "Temporal Dynamics" OR "Coordinat*" OR "Interpersonal Coordinat*" OR "Social* Coordinat*" OR "Motor Coordination" OR “Bod* Coordination" OR "Rhythm*") |
| Limiters: publication type | AND [excluding] DOCUMENT TYPES: (Editorial Material OR Correction OR Meeting Abstract OR Book Review OR News Item) |
| Limiters: subject area | [excluding] WEB OF SCIENCE CATEGORIES: ( Neurosciences OR  Computer Science Interdisciplinary Applications OR Toxicology OR Immunology OR Microbiology OR Nanoscience Nanotechnology Or Sport Sciences OR Audiology Speech Language Pathology OR Chemistry Multidisciplinary OR Acoustics OR Cell Tissue Engineering OR Chemistry Inorganic Nuclear OR Instruments Instrumentation OR Nursing OR Biochemical Research Methods OR Engineering Electrical Electronic OR Plant Sciences OR Biochemistry Molecular Biology OR Genetics Heredity OR Cardiac Cardiovascular Systems OR Materials Science Multidisciplinary OR Anatomy Morphology OR Computer Science Artificial Intelligence OR Surgery OR Electrochemistry OR Health Policy Services OR Engineering Industrial OR Physiology OR Telecommunications OR Environmental Sciences OR Cell Biology OR Chemistry Physical OR Energy Fuels OR Integrative Complementary Medicine OR Pharmacology Pharmacy OR Engineering Multidisciplinary OR Nutrition Dietetics OR Radiology Nuclear Medicine Medical Imaging OR Oncology OR Ophthalmology OR Optics OR Otorhinolaryngology OR Medicine General Internal OR Computer Science Hardware Architecture OR Pathology OR Endocrinology Metabolism OR Physics Applied OR Zoology OR Chemistry Analytical OR Engineering Biomedical OR Spectroscopy OR Engineering Mechanical OR Biotechnology Applied Microbiology OR Hematology OR Computer Science Software Engineering OR Infectious Diseases OR Engineering Chemical OR Public Environmental Occupational Health OR Imaging Science Photographic Technology OR Obstetrics Gynecology OR Respiratory System OR Orthopedic) |
| Limiters: research area | [excluding] RESEARCH AREAS: (Business Economics OR Dentistry Oral Surgery Medicine OR Dermatology OR Environmental Sciences Ecology OR General Internal Medicine OR Forestry OR History Philosophy of Science OR Geochemistry Geophysics OR Information Science Library Science OR Geology OR Geriatrics Gerontology OR Medical Laboratory Technology OR Oceanography OR Physical Geography OR Pharmacology Pharmacy OR Physics OR Engineering OR Virology OR Rheumatology OR Art OR Philosophy OR Transportation OR Agriculture OR Astronomy Astrophysics OR Urology Nephrology OR Anesthesiology OR Chemistry OR Veterinary Sciences OR Anthropology OR Construction Building Technology OR Water Resources) |

### PsycArticles and PsycINFO

| ASC search terms | (TITLE-ABS-KEY ( "Autism Spectrum Disorder*" OR "Autism Spectrum Condition*" OR autis* OR "ASD" OR "ASC" OR "Autis* Traits" OR "Autis* Characteristics" OR asperger* ) |
| --- | --- |
| SMS search terms | AND TITLE-ABS-KEY ( "Social Motor Sync*" OR "Interpersonal Sync*" OR sync* OR "Motor Sync*" OR "Interactional Sync*" OR "Social Sync*" OR "Behavioural Sync*" OR "Behavioral Sync*" OR "Temporal Sync*" OR "Rhythmic Movment" OR "Temporal Dynamics" OR "Coordinat*" OR "Interpersonal Coordinat*" OR "Social* Coordinat*" OR "Motor Coordination" OR "Bod* Coordination" OR "Rhythm*" OR “Interpersonal Coordinat*” OR “Social Coordinat*” OR “Motor Coordinat*” OR “Bod* Coordinat*”) ) |
| Limiters | **Record type:** Journal, Journal Article, Peer Reviewed Journal, Full-Text, Peer-reviewed  **Language:** English  **Population:** Human |

### PubMed

| ASC search terms | (TITLE-ABS-KEY ( "Autism Spectrum Disorder*" OR "Autism Spectrum Condition*" OR autis* OR "ASD" OR "ASC" OR "Autis* Traits" OR "Autis* Characteristics" OR asperger* ) |
| --- | --- |
| SMS search terms | AND TITLE-ABS-KEY ( "Social Motor Sync*" OR "Interpersonal Sync*" OR sync* OR "Motor Sync*" OR "Interactional Sync*" OR "Social Sync*" OR "Behavioural Sync*" OR "Behavioral Sync*" OR "Temporal Sync*" OR "Rhythmic Movment" OR "Temporal Dynamics" OR "Coordinat*" OR "Interpersonal Coordinat*" OR "Social* Coordinat*" OR "Motor Coordination" OR "Bod* Coordination" OR "Rhythm*" OR “Interpersonal Coordinat*” OR “Social Coordinat*” OR “Motor Coordinat*” OR “Bod* Coordinat*”) |
| Limiters | **Language:** English  **Population:** Human |
